# Competing risks and the clinical community: irrelevance or ignorance?

**DOI:** 10.1002/sim.4384

**Published:** 2011-09-23

**Authors:** Michael T Koller, Heike Raatz, Ewout W Steyerberg, Marcel Wolbers

**Affiliations:** a Basel Institute for Clinical Epidemiology and Biostatistics, University Hospital BaselSwitzerland; b Clinic for Transplantation Immunology and Nephrology, University Hospital BaselSwitzerland; c Departments of Public Health, Erasmus MCRotterdam, The Netherlands; d Wellcome Trust Major Overseas Programme and Oxford University Clinical Research UnitHo Chi Minh City, Vietnam

**Keywords:** competing risks, clinical interpretation, quality of reporting, cause-specific hazard, subdistribution hazard

## Abstract

Life expectancy has dramatically increased in industrialized nations over the last 200 hundred years. The aging of populations carries over to clinical research and leads to an increasing representation of elderly and multimorbid individuals in study populations. Clinical research in these populations is complicated by the fact that individuals are likely to experience several potential disease endpoints that prevent some disease-specific endpoint of interest from occurrence. Large developments in competing risks methodology have been achieved over the last decades, but we assume that recognition of competing risks in the clinical community is still marginal. It is the aim of this article to address translational aspects of competing risks to the clinical community. We describe clinical populations where competing risks issues may arise. We then discuss the importance of agreement between the competing risks methodology and the study aim, in particular the distinction between etiologic and prognostic research questions. In a review of 50 clinical studies performed in individuals susceptible to competing risks published in high-impact clinical journals, we found competing risks issues in 70% of all articles. Better recognition of issues related to competing risks and of statistical methods that deal with competing risks in accordance with the aim of the study is needed. Copyright © 2011 John Wiley & Sons, Ltd.

## 1 Introduction

Competing risks are an extension of classical survival analysis. In competing risks, we observe different event types in addition to the time to the first event occurring, possibly subject to censoring or left truncation. In medical research, competing risks occur when the time to a disease-specific endpoint of interest may be precluded by death or a major health event from another cause. The statistical methodology for analyzing competing risks data has rapidly expanded over the last decades. However, clinical studies often ignore competing risks or the multistate process of clinical endpoint generation and there appears to be a limited awareness of the importance and pitfalls of competing risks in the clinical community 1.

The aim of this work is therefore to address translational aspects of competing risks to the clinical community. We first describe study populations susceptible to competing risks. These rapidly growing populations highlight the increasing need for competing risks approaches in clinical research. After a short overview of competing risks concepts, we show how competing risks interfere with the understanding of diseases in clinical medicine and epidemiology. Moreover, we assess the popularity of the topic of competing risks in biostatistical and clinical journals based on a literature search. Finally, we critically appraise the quality of reporting competing risks in studies performed in populations susceptible to competing risks that were published in high-ranked clinical journals.

## 2 The population susceptible to competing risks

Over the last 200 years, life expectancy has dramatically increased in industrialized countries. In Norway, for example, life expectancy in 1866 was 47 and 51 years in males and females, respectively, and has linearly increased to 79 and 83 years in 2000 2. The age distribution has thus rapidly changed with an increasing proportion of elderly subjects in populations of industrialized countries.

The aging of populations has led to an increase of diseases attributable to aging and frailty such as cancer, chronic heart failure or dementia. Moreover, it was shown in the US that the majority of healthcare resources are spent in people older than 60 years 3, and that the largest fraction of a person's total health costs arises during the last months of life 4. The aging of populations has consequences for clinical research. Study populations of common diseases increasingly consist of elderly individuals with varying degrees of multimorbidity 5. If the aim is to observe the time to some disease-specific endpoint of interest, such study participants should be considered as susceptible to competing risks.

More precisely, the following characterization identifies study populations susceptible to competing risks:

1. Individuals are likely to host several yet unrecognized diseases or they are at risk to develop more than one disease that all may lead to clinically relevant competing disease endpoints.
2. The first occurrence of such an endpoint has a strong impact on the individual. Examples are death, cerebral stroke with handicap, heart failure after a heart attack with limitations of physical activity and need for regular pharmacological therapy.

Periods of susceptibility to competing risks are typically late in life with increasing disease accumulation and frailty. Less prevalent but equally relevant are periods of critical or severe illness 6. Table 1 summarizes the characteristics of these two distinct presentations of susceptibility.

**Table 1:** Characteristics of two typical patient populations susceptible to competing risks.

The elderly/multimorbid	The critically/severe ill
‘(Slowly) progressive biology’	‘Destructing biology’
Long-term risk exposure, e.g. smoking, diabetes,hypertension, HIV	Short term risk exposure, e.g. acute infection, celldepletion, mechanical ventilation
Period: late life	Period: any
Examples: Elderly patients, chronic kidney disease, long-term diabetes or hypertension, HIV/AIDS,cardiovascular risk patient, prostate cancer patients	Examples: Intensive care unit patients, transplantrecipients, patients in aplasia, patients on chemotherapy

## 3 A concise overview of competing risks concepts

We restrict our short overview of competing risks concepts to two competing events and refer to Putter *et al*. 2007 for a more comprehensive tutorial. The observable data in competing risks is represented by the (possibly censored) time to the first event *T* and the cause of failure *D*, that is, either the event of interest (*D* = 1) or the competing event (*D* = 2). Two key concepts of competing risks, the cause-specific hazard and the cumulative incidence function (CIF), arise from describing different components of the transition of a patient from his baseline state to either failure cause.

**Figure 1:**
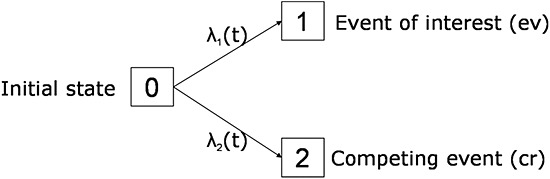
Competing risks as multistate model with cause-specific hazards *λ*_1_(*t*) and *λ*_2_(*t*).

The *cause-specific hazards view* describes the competing risks process as a multistate model with initial state 0 and two absorbing states 1 (event of interest) and 2 (competing event, Figure 1) 8. The transition intensity from the initial state to either of the absorbing states is determined by the cause-specific hazards *λ*_1_(*t*) and *λ*_2_(*t*) defined as 

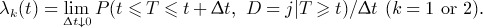


The cause-specific hazards can be interpreted as the momentary forces that draw a subject out of state 0 into state 1 or 2 9. As the cause-specific hazards definition conditions on *T ≥ t*, all patients with failure (of any cause) before time *t* are removed from the risk set at that time point. Proportional cause-specific hazards regression models can be estimated using software for standard Cox regression and censoring patients with competing events at the time point of their occurrence 7.

The CIF describes the actual (absolute) risk of failing from cause *k* until time *t* : *I*_*k*_(*t*) = *P*(*T ≤ t* and *D* = *k*). A graphical display of the estimated CIFs for all competing events serves as a key summary of the competing risks process (similar to the Kaplan–Meier curve in survival analysis 7). The CIFs are determined by the cause-specific hazards but, importantly, they depend not only on the cause-specific hazard of the respective event but also on the total hazard through the relation 10

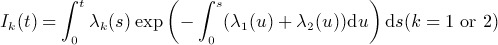


**Figure 2:**
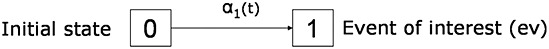
Competing risks model with the subdistribution hazards approach *α*_1_(*t*).

This complicates the interpretation of regression models for cause-specific hazards on the cumulative incidence scale 11. To avoid this complication, Fine and Gray 1999 introduced a way to regress directly on CIFs by specifying a proportional hazards model for the so-called subdistribution hazards defined as *α*_*k*_(*t*) : = −*d*log(1 −*I*_*k*_(*t*))/d*t* (Figure 2). Importantly, this approach uses a revised definition of the risk set where persons who fail from other causes remain in the risk set and act as placeholder for those who will never experience the event of interest 12,13.

There has also been interest in the *marginal survival distribution* of the event of interest, that is, the survival time in a virtual world where competing events do not exist. Unlike the cause-specific hazards and the CIF, the marginal distribution is not identifiable from competing risks data 14 and depends on the exact means of removal of the competing causes. Greenland 2002 illustrated this for overall survival in a virtual world where death from lung cancer did not exist: The marginal survival curve in this virtual world would be expected to be quite different depending on whether the cause-removal of lung cancer deaths was because of a new highly effective chemotherapy (which does not affect other death causes) or a comprehensive behavioural intervention including smoking cessation (which would also reduce other death causes such as coronary heart disease or chronic obstructive pulmonary disease).

In clinical manuscripts, marginal survival estimates are often based on treating competing events as censoring events and calculating Kaplan–Meier estimates (Section 5). Importantly, these estimates are valid only under the assumption of independence of the (latent) marginal failure times for different causes 7. If we use survival estimates based on standard Cox regression instead, independence conditional on covariates must be assumed. In both cases, the independence assumption cannot be tested and is often *a priori* unrealistic. Marginal survival estimates in the clinical literature should thus be interpreted with caution, in particular if the means of removal of competing causes is not clearly defined or implicit independence assumptions are made without clinical justification. Finally, many publications have shown that the Kaplan–Meier estimator overestimates the CIF in the presence of competing risks and should therefore not be used for this purpose 16.

As for survival data, left-truncation and interval censoring may also occur for competing risks data. It is straightforward to account for left-truncation in cause-specific hazards models 7 and a method for fitting the Fine and Gray model to left-truncated and right-censored data has recently been suggested 17. Interval censored competing risks data are more challenging to analyze; one valid approach is to use parametric competing risks models 18,19.

Finally, a commonly adopted method to circumvent competing risks issues is the use of a composite endpoint that combines all-cause mortality and a disease-specific failure endpoint of interest 20. Often, however, in drug efficacy research or during the investigation of causal pathways, investigators may also wish to assess the effect of exposure on the disease-specific endpoint of interest rather than on the composite endpoint.

## 4 How do competing risks interfere with our understanding of disease?

Today's concepts of understanding diseases go back to Hippocrates. He understood diseases in terms of diagnosis, etiology (what is the cause of a disease?), treatment and prognosis. These concepts have been inherent in the formation and thinking of clinicians for centuries 21 and have become fundamental to the thinking in epidemiology 22. It is thus of interest to discuss how competing risks affect this basic understanding of diseases.

The concept of disease diagnosis is not of much interest in regard to competing risks and will not be discussed further. The treatment concept is analogous to the etiology concept because treatments exert their effects by modifying causal pathways of disease development. In clinical studies treatment effects are thus often referred to as efficacy. Thus, two distinct concepts remain to be discussed, etiology/efficacy and prognosis.

We believe that etiology/efficacy hypotheses in the presence of competing risks are most naturally formulated in terms of cause-specific hazards. This allows for a ‘direct’ formulation of the effect of exposure on the instantaneous forces that drive the patients remaining at risk at each time point *t*, that is, those without any prior event. On the other hand, the absolute risk of events occurring over time is the natural basis for prognosis and medical decision making 23 and this can be assessed most conveniently by models that directly regress on the CIF 24,26.

Many efforts have been made to give etiologic effect measures a clinically more intuitive interpretation. To do so, measures of etiologic associations are translated into measures of prognosis. Two well-known examples are the average duration of life gained 27 and the number needed to treat 28. For survival endpoints without competing risks, the translation of the etiologic effect (relative change in the hazard) into prognosis (change in survival) is unambiguous as an increased hazard has a 1–1 association with a shorter survival time. This association only breaks down in the presence of nonproportional hazards, that is, treatment-time interactions. However, with competing risks, this translation is generally ambiguous as we illustrate below.

**Figure 3:**
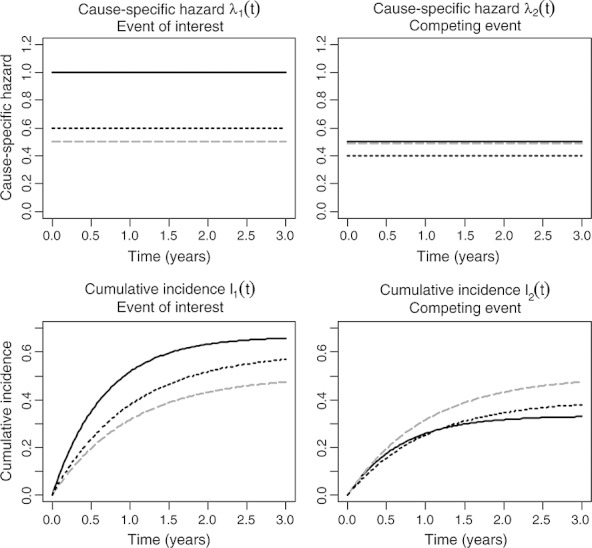
Hypothetical three-arm trial with a competing risks outcome. The top two panels show the assumed cause-specific hazards for placebo (solid black lines) and treatment 1 (dashed gray lines), which affects only the cause-specific hazard of the event of interest and treatment 2 (dotted black lines), which affects both cause-specific hazards. The two bottom panels show the resulting cumulative incidence functions.

We use a hypothetical perfectly conducted three-arm randomized trial of placebo versus two treatments for our illustration. The endpoint is the time to a cause-specific mortality (e.g. death from lung cancer) as the event of interest and death from all other causes as the competing event. We assume constant cause-specific hazards *λ*_1_ and *λ*_2_ for the two events, respectively. Under this assumption, it can easily be shown that the CIF for the event of interest is given by *I*_1_(*t*) = *λ*_1_/(*λ*_1_ + *λ*_2_) ⋅(1 −exp(−(*λ*_1_ + *λ*_2_) ⋅*t*) (and similarly for the competing risk). We further assume that the cause-specific hazards under placebo are 1 and 0.5, respectively, and that treatments act proportionately on the cause-specific hazards. Treatment 1 is efficacious and reduces the hazard of the event of interest by 50% but does not affect the hazard of the competing event; whereas treatment 2 reduces both hazards by 40% and 20%, respectively. The assumed cause-specific hazards and the resulting CIFs are displayed in Figure 3.

The figure shows an increase in the cumulative incidence of the competing risk for treatment 1 even though etiologically, treatment 1 does not affect the competing event. Even under treatment 2, which also moderately reduces the cause-specific hazard of the competing event, the cumulative incidence is ultimately increased. Therefore, the changes in the CIF reflect not only the true effect of treatment on the competing event (there was none for treatment 1) but also all modifications of the risk set caused by the effect of treatment on the event of interest. More specifically, in this example, treatment prolonged the life of the hypothetical subjects by reducing the occurrence of the more likely event of interest, which increased the risk set of patients susceptible to competing death causes 29. Because we assumed a perfectly conducted randomized trial, there is a causal treatment effect on both the cause-specific hazard and the CIF. However, the causal effects on the cause-specific hazards are much easier to interpret etiologically whereas the CIF is a pragmatic quantity.

## 5 Competing risks issues in the clinical literature

**Figure 4:**
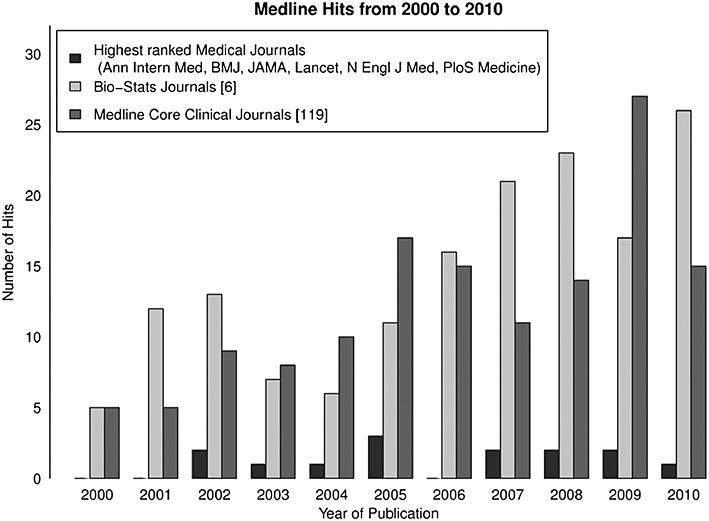
Absolute number of articles published on competing risks between 1 January 2000, and 28 October 2010, in the highest ranked clinical journals, biostatistical journals, and core clinical journals.

We first assessed the frequency of studies published on the subject of competing risks within the last 10 years in three different fields: biostatistical journals (six journals: Biom J, Biometrics, Biostatistics, Lifetime Data Anal, Stat Methods Med Res, Stat Med), core clinical journals (119 different journals) and the six general clinical journals with the highest impact factors (Ann Intern Med, BMJ, JAMA, Lancet, N Engl J Med and PloS Medicine, see Appendix A for a detailed search strategy). Figure 4 shows the absolute number of articles published over the last 10 years, which steadily increased over time with a maximum of 27 articles in core clinical journals in 2009 and roughly a similar number of publications per year in the six biostatistical and the 119 core clinical journals. In the high-impact medical journals, only between 0 and 3 articles including the topic competing risks were published per year.

Then we examined how competing risks issues were treated in high-impact medical journals. We therefore performed a systematic Medline search of scientific articles published in the last 3 years (i.e. from 28 October 2007, until 28 October 2010) in those journals in the setting of populations susceptible to competing risks. We defined a number of diseases that are prevalent or typical in aging or multimorbid patients and where follow-up studies potentially had competing risks issues (see Appendix A for detailed search strategy). We screened the articles and included those studies where one or more time-to-event endpoints were reported and analyzed. Exclusion criteria were: all-cause mortality was the only endpoint under investigation; only short-term course of disease studied; case report, case-control, or ecological studies.

For an in-depth review, we included the latest 50 articles fulfilling the inclusion/exclusion criteria. Two reviewers (HR and MK) critically appraised all 50 included articles.

For each included study, we scrutinized the endpoint definitions and identified competing risks as: (i) competing death — if the endpoint of interest was some nonfatal disease-specific endpoint (e.g. nonfatal stroke) or (ii) ‘other causes of death’ — if the endpoint of interest was some disease-specific death (e.g. prostate cancer related death) or a combined endpoint of fatal and nonfatal disease-specific events. For simplicity, we only defined the absorbing state of ‘competing death’ or ‘other causes of death’ as competing risk events.

**Figure 5:**
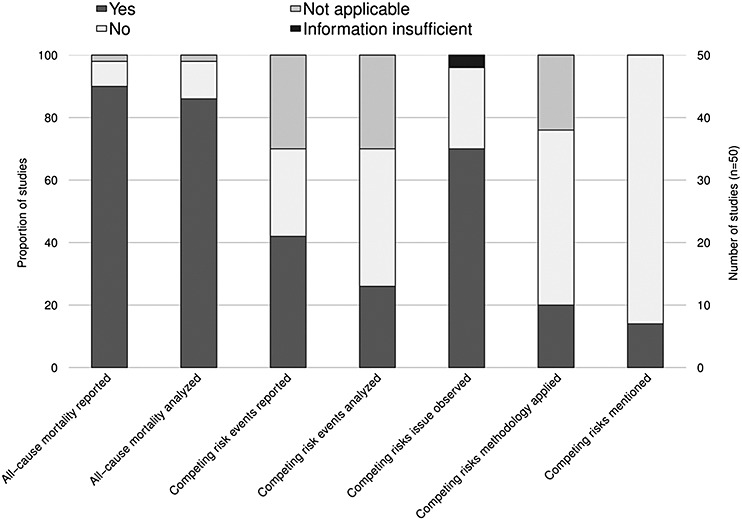
Critical appraisal for competing risks issues in 50 studies published in high-ranked clinical journals and populations susceptible to competing risks.

If competing risks were present, we defined competing risks issues as (Figure 5):

1. Estimation of marginal survival curves based on the Kaplan–Meier estimator (‘naive Kaplan–Meier estimate’). Such estimates are sometimes referred to as ‘cause-specific survival’ 30 or death-censored survival 31 and, as described at the end of Section 3, are interpretable only under strong independence assumptions.
2. The competing risks process was neglected: (i) competing death not reported or (ii) crude number of competing events reported but not analyzed

We moreover extracted the following data from the included studies: reporting and/or analysis of all-cause mortality; reporting and/or analysis of competing risk events; application of competing risks methodology with details on the method used; and whether competing risks were mentioned or discussed in the article (Figure 5).

Results: In 74% (37/50) of all included studies, the definition of at least one endpoint implied the presence of competing risks; the remaining 13 studies exclusively defined composite endpoints with all-cause mortality as component. In 70% (35/50) of the included studies, we actually observed at least one competing risks issue: Naive Kaplan–Meier estimates in the presence of competing risks in 67% (24/35) and complete ignorance of competing risks in 51% (18/35) of the studies. Although 90% (45/50) of the included studies reported (a composite endpoint of) all-cause mortality among other endpoints, 67% (30/45) of these studies nevertheless revealed competing risks issues during the analysis of disease-specific endpoints (Figure 5). Moreover, 4 of the 13 studies that exclusively defined a composite endpoint of all-cause mortality showed competing risk issues at some point during the analysis.

Only 20% (10/50) of the included studies explicitly applied competing risks methodology and 18% (9/50) discussed competing risks as a possible issue (Figure 5). The competing risks methodology most frequently applied (in 8/50 studies) was the analysis of *all* cause-specific hazards corresponding to the competing risks process. However, half of these 10 studies nevertheless presented survival curves based on naive Kaplan–Meier estimates. Only two studies explicitly used the CIF in place of (biased) cumulative incidence estimates based on the Kaplan–Meier method.

## 6 Discussion

We defined study populations susceptible to competing risks and critically appraised 50 recent studies in these populations published in high-impact medical journals. We found that a large proportion of studies analyzed endpoints that are subject to competing risks but that competing risks issues were often ignored and that the application of inappropriate statistical methods was a frequent problem.

However, competing risks are clearly not irrelevant for medical research and their negligence has important clinical consequences: first, the naive interpretation of Kaplan–Meier estimates in the presence of competing risks as estimates of actual risks leads to potential overestimation of the benefit and cost-effectiveness of interventions in clinical trials 32,33 and overestimation and inappropriate risk stratification in prognostic models 24. This may result in over-treatment with potential harmful consequences for individuals and healthcare systems. Second, disregarding competing risks masks the fact that in some populations the incidence of competing events may be much larger than the incidence of the event of interest 24, which limits the clinical usefulness of costly interventions to reduce some disease-specific endpoint of interest. In particular, this may occur if indications for interventions tested in clinical trials with rigid inclusion/exclusion criteria are expanded to more frail populations 30,31. Third, analyzing only part of the multistate model describing the competing risks data may miss important insights into diseases and unexpected effects of interventions on disease-unrelated causes such as effects of antiretroviral therapy on non-AIDS-related deaths 34.

Even if competing risks are acknowledged, a choice between different statistical techniques has to be made. We illustrated with a simple example that the one-to-one association between etiology and prognosis that holds for traditional survival models breaks down in the presence of competing risks and that this interferes with the traditional understanding of diseases. As we have shown, an intervention may not etiologically affect the cause-specific hazard of an event of interest but still alter the actual risk of this event through the indirect effects on the cause-specific hazards of competing events. The CIF therefore reflects a pragmatic, but not necessarily a biologic quantity. In line with other authors 13, we believe that the context of the research question is the main determinant for the choice of an appropriate statistical model. For etiologic questions, cause-specific hazards models are generally more appropriate. In contrast, if the focus is on the direct assessment of actual risks and therefore prognosis and medical decision making, regression models for the CIF are preferred.

## 7 Limitations

First, there are several fields in medical research that were not discussed but where competing risks play an important role. This includes competing beneficial and harmful events (e.g. hospital discharge from ICU and death in ICU) 6,11,33 and the field of device or transplant survival estimation, where it has been heavily debated how recipients' deaths should be adequately accounted for 34,35. Second, we have not discussed the implications of violations of model assumptions such as nonproportional cause-specific or subdistribution hazards. Mathematically, proportional hazards can only hold for either the cause-specific or the subdistribution hazards, but it has been shown that proportional subdistribution hazard modelling offers a summary analysis, even if mis-specified 35. Nevertheless, goodness-of-fit may also impact on the choice of the competing risks model. Third, in some competing risks applications, not all competing events are fatal and there are also potential transitions between failure types. In this case, a more general illness-death or multistate model would be required for a comprehensive analysis 7. Finally, we discussed the distinction between etiology and prognosis but did not attempt a comprehensive discussion of the important topic of causality in competing risks models.

## 8 Conclusion

The increasing consideration of populations susceptible to competing risks in clinical studies highlights the need for competing risks approaches in clinical research. The key conceptual differences in the application of competing risks methodology pertain to etiologic and prognostic questions. A review of clinical studies in frail populations published in high-ranked clinical journals showed competing risks issues in 70% of all articles. A better recognition of competing risks in the clinical community is needed.

## A. Appendix

Pubmed search strategy for studies on competing risks in biostatistical journals:(‘competing cause’ OR ‘competing causes’ OR ‘competing risk’ OR ‘competing risks’ OR ‘competing outcome’ OR ‘competing outcomes’ OR ‘competing endpoints’) AND (Biom J[Jour] OR Biometrics[Jour] OR Biostatistics[Jour] OR Lifetime Data Anal[Jour] OR Stat Methods Med Res[Jour] OR Stat Med[Jour])

Limits activated: English, Field: Title/Abstract, published in the last 10 yearsSearched time period: 1 January 2000 until 28 October 2010

Pubmed search for studies of patients susceptible to competing risks in high-impact medical journals:(atrial fibrillation OR cardiac failure OR coronary heart disease OR stroke OR aneurysm OR ((prostate OR colorectal OR breast) AND cancer) OR chronic leukemia OR cancer screening OR critical care OR transplant* OR chronic obstructive pulmonary disease) AND (N Engl J Med[Jour] OR JAMA[Jour] OR bmj[Jour] OR Ann Intern Med[Jour] OR Lancet[Jour] OR PLoS Med[Jour]) AND (survival OR mortality) NOT retrospective NOT case-control study NOT short-term NOT in-hospital NOT 30-day

Limits activated: only items with abstracts, Humans, English, Aged: 65+ years, published in the last 3 years.
